# Navigating Mirror Anatomy: Successful Percutaneous Endoscopic Gastrojejunostomy Tube Placement in Situs Inversus

**DOI:** 10.7759/cureus.109192

**Published:** 2026-05-19

**Authors:** Monika Stoskute, Rangesh Modi, Edwin K McDonald, Yichun Fu

**Affiliations:** 1 Internal Medicine, University of Chicago, Chicago, USA; 2 Gastroenterology, Hepatology, and Nutrition, University of Chicago, Chicago, USA

**Keywords:** endoscopic techniques, enteral nutrition, pegj, percutaneous endoscopic gastrojejunostomy tube, situs inversus

## Abstract

Situs inversus (SI) is a rare congenital laterality disorder characterized by complete mirror-image reversal of the thoracoabdominal organs. Percutaneous endoscopic gastrostomy with jejunal extension is commonly indicated for postpyloric enteral nutrition but has not previously been described in patients with SI. We report a case of successful bedside percutaneous endoscopic gastrojejunostomy (PEGJ) tube placement in a 60-year-old woman with primary ciliary dyskinesia and complete SI following bilateral lung transplantation. Preprocedural imaging confirmed mirror-image anatomy. PEGJ tube placement was performed endoscopically using a gastrostomy tube with jejunal extension and required technical modifications to accommodate the reversed anatomy. We demonstrate that PEGJ tube placement in patients with SI is feasible and safe with appropriate preprocedural planning and adaptive endoscopic techniques. Awareness of the altered anatomy, careful site selection, and flexibility in tube advancement strategies are essential to minimize complications and ensure procedural success.

## Introduction

In this case, we describe percutaneous endoscopic gastrojejunostomy (PEGJ) tube placement in a patient with situs inversus (SI) in the setting of primary ciliary dyskinesia (PCD). 

About situs inversus 

SI is a human laterality disorder defined by the complete mirror-image reversal of the thoracoabdominal organs across the left-right axis [1]. Situs solitus, on the other hand, describes normal organ arrangement [2]. Between situs solitus and SI lies a spectrum of anatomical laterality changes termed situs ambiguus, also known as heterotaxy [3].

The birth prevalence of SI is estimated to be 1.1 per 10,000 live births (95% CI, 1.0-1.2) [4]. SI is often found incidentally, as it is usually asymptomatic unless associated with conditions such as PCD or congenital heart disease. PCD, formerly known as Kartagener syndrome, is a disorder of motile cilia causing impaired mucociliary clearance and typically presents as a triad of SI, chronic bronchiectasis, and sinusitis. The rate of lung transplantation in patients with PCD is typically less than 5% [5].

The diagnosis of SI is particularly important for surgical and procedural planning. Case reports have described various surgical procedures in patients with SI, including gastrointestinal procedures such as endoscopic retrograde cholangiopancreatography (ERCP) and percutaneous endoscopic gastrostomy (PEG) placement. Regarding PEG placement, several case reports have demonstrated procedural success, with the suggestion of positioning patients with SI in the left lateral decubitus position [6,7]. Some patients with SI, however, may require PEGJ, which is more technically complex than PEG placement. Combined with mirror-image anatomy, this may introduce challenges for endoscopic navigation. To our knowledge, no studies have been published on PEGJ placement in patients with SI. Here, we describe a case of PEGJ tube placement in a patient with SI in the setting of PCD.

## Case presentation

The patient is a 60-year-old female with a past medical history of PCD with complete SI, severe bronchiectasis, chronic hypoxemic respiratory failure, type 2 diabetes mellitus, and generalized tonic-clonic seizures, who underwent elective bilateral lung transplantation and tracheostomy due to prolonged critical illness. She had no prior intra-abdominal surgeries. Prior to admission, the patient had no gastrointestinal symptoms and was tolerating 100% of oral intake at baseline. However, she had a body mass index of 19 and sarcopenia secondary to hypermetabolism from chronic lung disease. She developed persistent abdominal distension and high gastric residual volumes with nasogastric tube feeding, likely due to gastroparesis secondary to vagal nerve disruption during lung transplantation. Given intolerance to gastric tube feeding, a nasojejunal (NJ) tube was placed endoscopically, and she tolerated jejunal feeding. However, due to persistent ventilator dependence, she required long-term enteral access. She had no ascites, coagulopathy, or prior gastric surgery. PEGJ placement was successfully performed by our gastroenterology team at the bedside in the intensive care unit. The procedural description is provided below.

Procedure description

The anatomy was reviewed on abdominal computed tomography (CT) imaging, which confirmed SI, with the liver in the left upper quadrant and the stomach extending toward the midline and right upper quadrant (Figure 1). Tube feeds were held for 12 hours prior to the procedure. The patient was placed in the supine position under standard sterile conditions, and a preprocedural timeout was performed.

**Figure 1:**
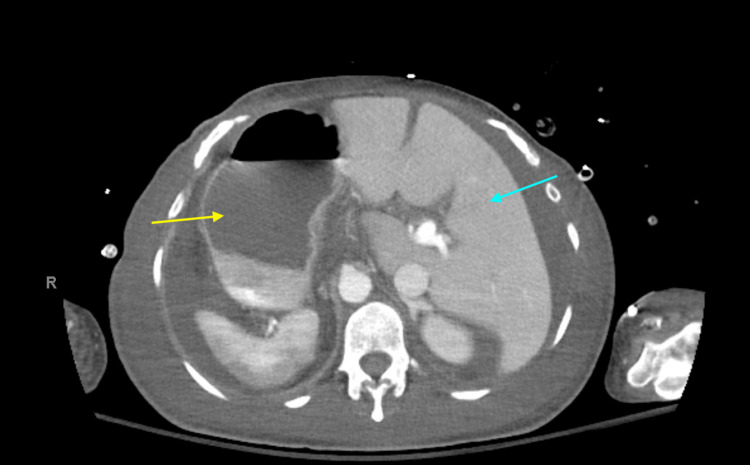
Computed tomography showing situs inversus The yellow arrow indicates the stomach, and the blue arrow indicates the liver.

One gram of cefazolin was administered as a prophylactic antibiotic 30 minutes prior to the procedure. The previously placed NJ tube was removed manually. The procedure was performed by two physicians (one responsible for the surgical portion and the other for endoscopy) and one endoscopy technician.

A standard adult gastroscope was utilized for the esophagogastroduodenoscopy (EGD). The exam showed no oropharyngeal or esophageal abnormalities. There was a small amount of tube feed mixed with gastric secretions pooled at the fundus that was suctioned successfully. The stomach had normal-appearing mucosa, and the pyloric opening was patent (Figure 2). The duodenal sweep and the second portion of the duodenum were patent yet grossly angulated; the orientation was also opposite to the typical anatomy (Figure 3). 

**Figure 2:**
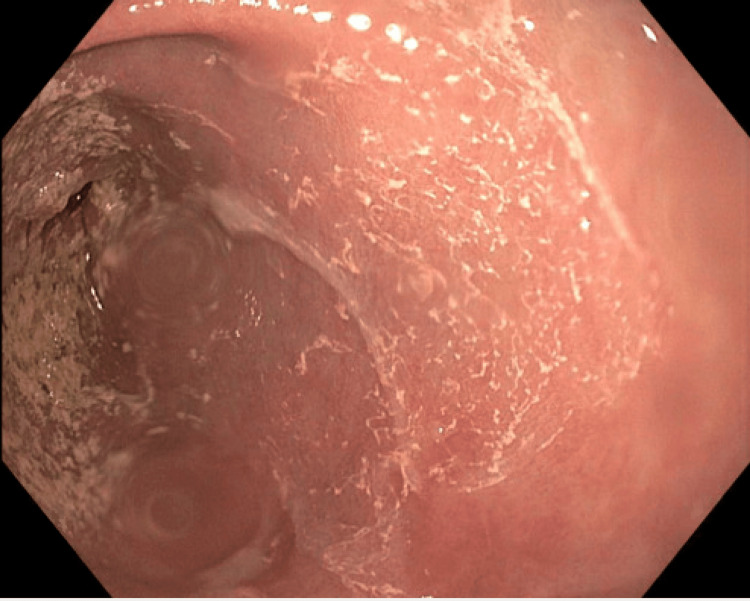
Endoscopic view of the stomach and pyloric opening

**Figure 3:**
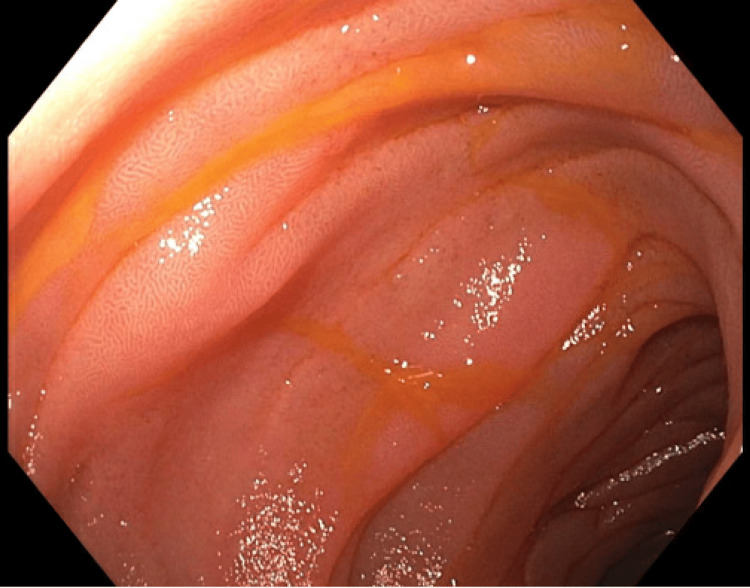
Endoscopic view of the duodenum

The endoscope was then withdrawn into the stomach, and the stomach was insufflated with carbon dioxide to oppose it to the abdominal wall. It was expected that the external point of maximal transillumination would be approximately 2 cm below the right costal margin in this patient with situs inversus; however, it was localized slightly left of midline, likely due to adequate gastric insufflation (Figure 4). One-to-one finger indentation was performed to identify the appropriate placement site and entry angle, helping avoid overlying loops of bowel or other organs. The internal site of maximal transillumination and indentation was located along the lesser curvature on the right side of the endoscopic view, instead of the usual left-sided orientation (Figure 5).

**Figure 4:**
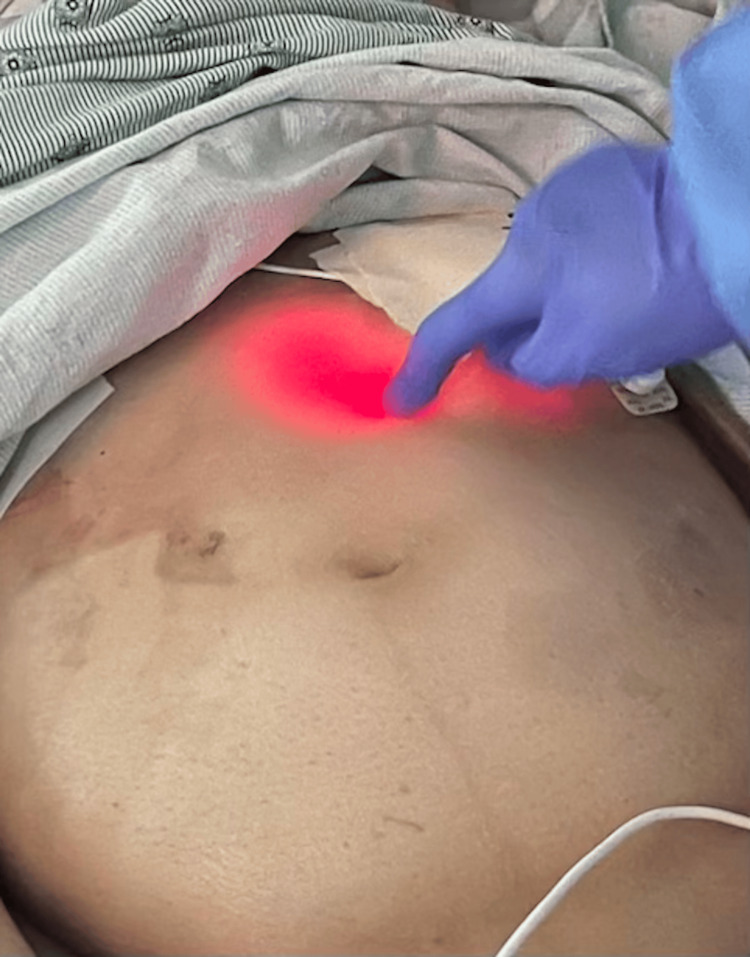
Point of maximal transillumination located slightly left of the midline

**Figure 5:**
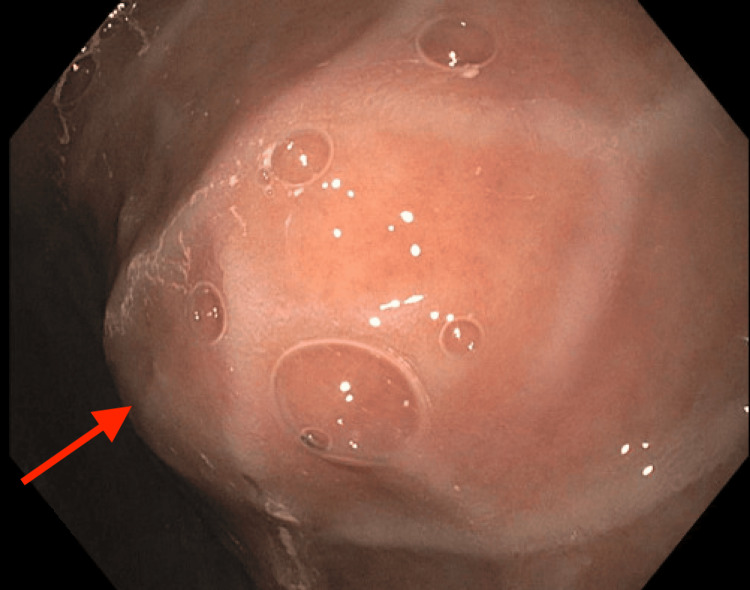
Identification of the appropriate placement site via endoscopic visualization of one-to-one finger indentation of the stomach (red arrow)

A 24-French (Boston Scientific, Marlborough, MA, USA) gastrostomy tube kit was opened. The skin surrounding our target site was cleaned with a generous amount of chlorhexidine. Five milliliters of 1% lidocaine was drawn into the lidocaine syringe needle provided in the kit. The needle was introduced at an acute angle into the skin at our target site, and a small wheal was created to provide local anesthesia. The needle was then gently introduced through the skin into the stomach while constantly pulling back on the syringe to confirm that the initial entry of air bubbles into the syringe coincided with visualization of the needle entering the stomach (safe track technique) (Figure 6). The needle was then withdrawn into the stomach mucosa, and the tract was anesthetized. 

**Figure 6:**
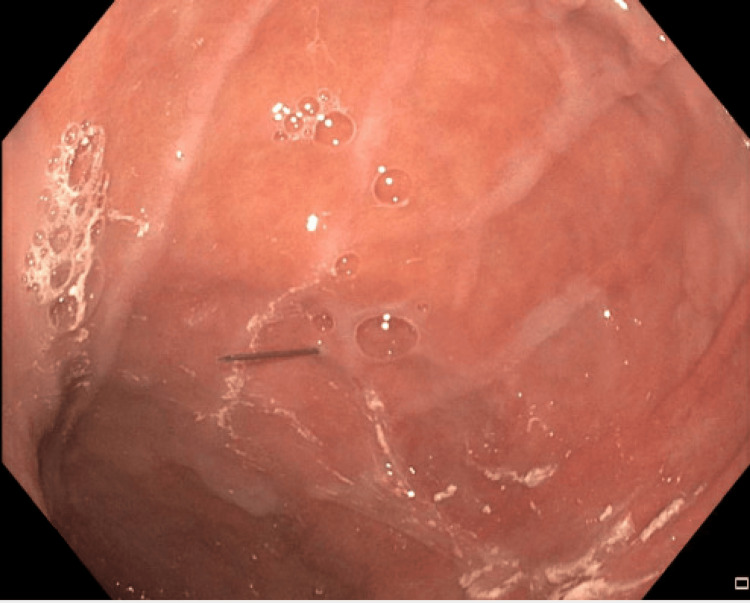
Needle entry using the safe track technique

A small incision was then made at the needle entry site with a scalpel, and a trocar was inserted into the stomach through the same tract, with care taken to maintain the same entry angle (Figure 7). At the same time, a snare was passed through the biopsy channel of the EGD scope. The trocar needle was withdrawn, and a guidewire was advanced through the trocar. The guidewire was then grasped with the snare, pulled out through the mouth, and secured to the 24-French gastrostomy tube. The gastrostomy tube was lubricated and pulled through the mouth, traversing the upper gastrointestinal tract, and brought out through the skin incision. The external bumper was secured at 2 cm, and endoscopy confirmed that the internal bumper was snug against the gastric wall with free mobility (Figure 8). The gastrostomy tube was then cut short, and a guidewire from the 12-French jejunal extension tube kit (Cook Medical, Bloomington, IN, USA) was introduced through the gastrostomy tube under endoscopic visualization (Figure 9). 

**Figure 7:**
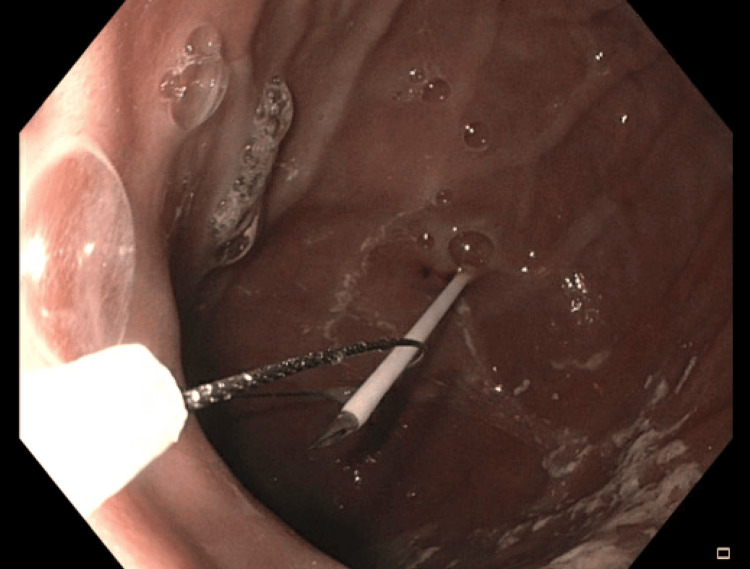
Trochar entry

**Figure 8:**
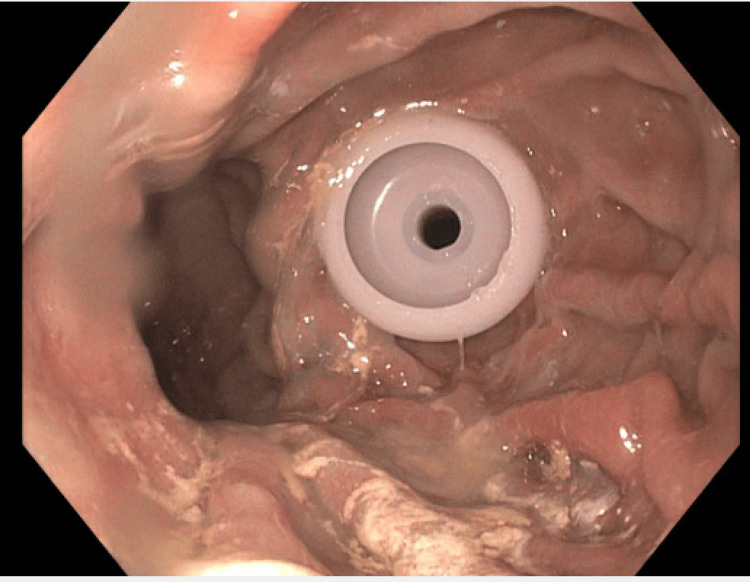
Endoscopic view of the internal bumper of the gastrostomy tube

**Figure 9:**
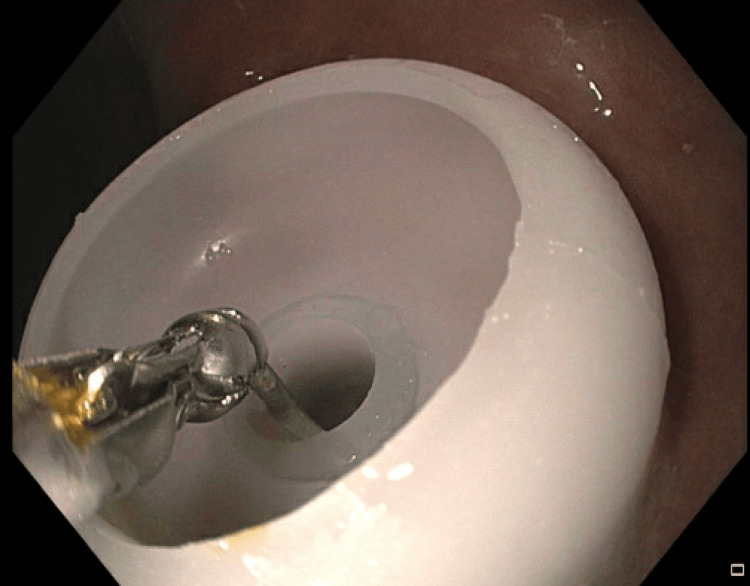
Guidewire introduced through the gastrostomy tube

An attempt to advance the guidewire into the small bowel using endoscopic rat-tooth forceps was unsuccessful due to the unusual orientation and angulation of the second and third portions of the duodenum (Figure 10). A nylon suture was tied in a loop fashion to the distal end of the 12-French jejunal extension tube, and the tube was lubricated. The guidewire was then completely withdrawn, and the jejunal extension tube was introduced into the stomach through the gastrostomy tube under endoscopic visualization (Figure 11). The suture at the end of the tube was grasped with rat-tooth forceps and pulled forward endoscopically (Figure 12). The tube was advanced slowly in several short increments, with a slight traction applied after each advancement to minimize gastric looping. 

**Figure 10:**
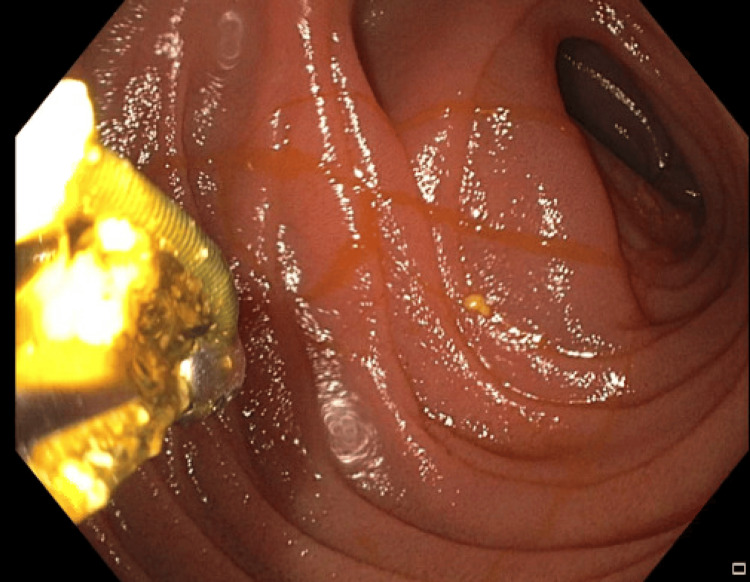
Attempt to advance the guidewire through the angulated duodenum

**Figure 11:**
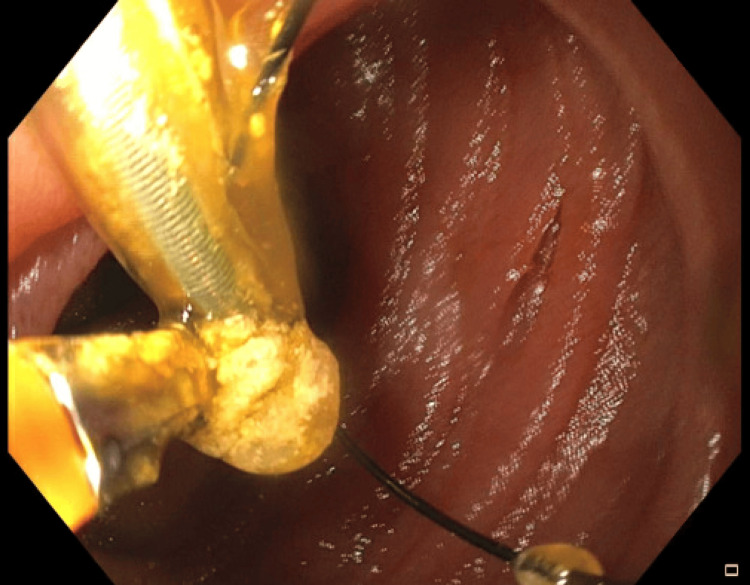
Jejunal extension tube introduced through the gastrostomy tube

**Figure 12:**
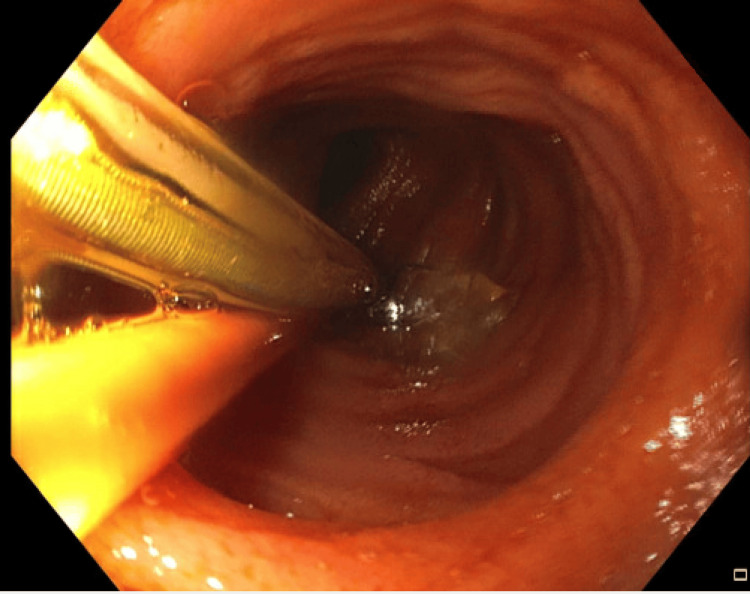
Sutured jejunal extension tube grasped with rat-tooth forceps and advanced through the small bowel under endoscopic guidance

We made an effort to maintain minimal tension within the small bowel lumen and optimize endoscopist ergonomics. When an external loop formed in the endoscope that increased tension, the loop was transferred toward the umbilicus by rotating the handpiece, using a technique commonly employed in colonoscopy loop reduction to maintain the scope in a neutral position. Once the tube reached the desired location in the jejunum, the rat-tooth forceps were withdrawn, and a clip was deployed through the scope. The suture was clipped to the intestinal wall to secure the tube in the small bowel (Figures 13-15). The scope was then withdrawn gently. The tube remained straight without evidence of looping during withdrawal. The total procedure time was 52 minutes. The patient tolerated the procedure well, with minimal blood loss limited to the initial skin incision (Figure 16). A postprocedural abdominal X-ray confirmed appropriate tube positioning (Figure 17). The patient subsequently tolerated jejunal tube feeds and demonstrated appropriate healing of the stoma site without leakage. A CT scan of the abdomen confirmed correct tube placement without pneumoperitoneum. The patient later expired from complications related to lung transplantation, including acute respiratory distress syndrome, sepsis, acute renal failure, and shock liver. 

**Figure 13:**
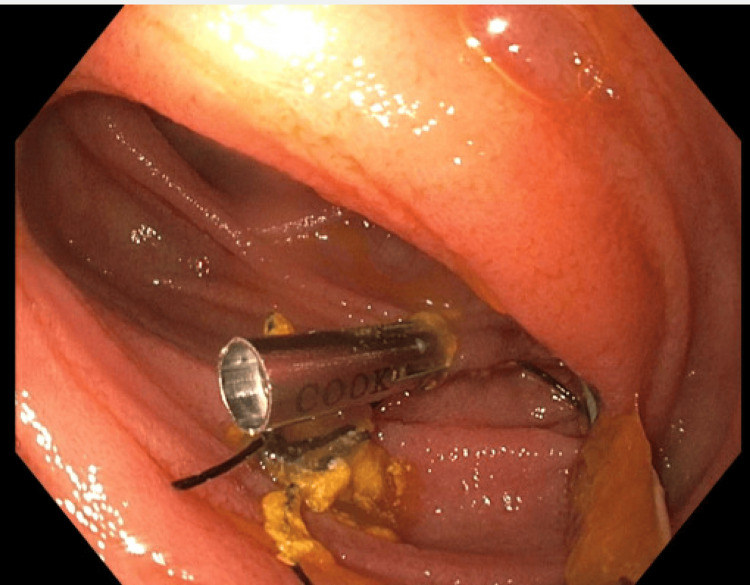
Suture attached to the jejunal extension tube and clipped to the small bowel

**Figure 14:**
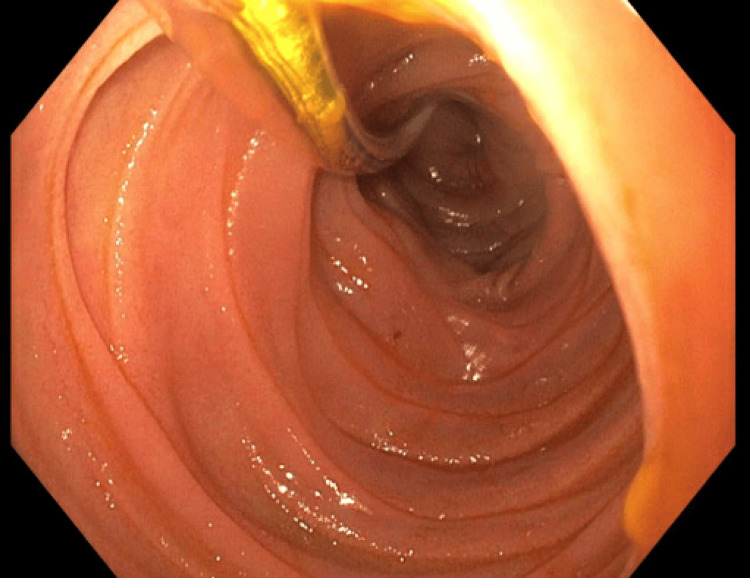
Jejunal extension tube secured within the small bowel

**Figure 15:**
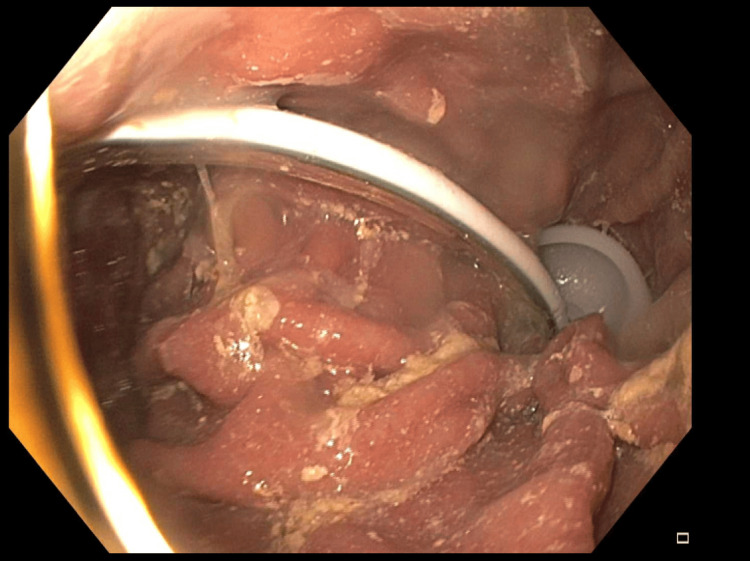
Path of the jejunal extension tube from the stomach

**Figure 16:**
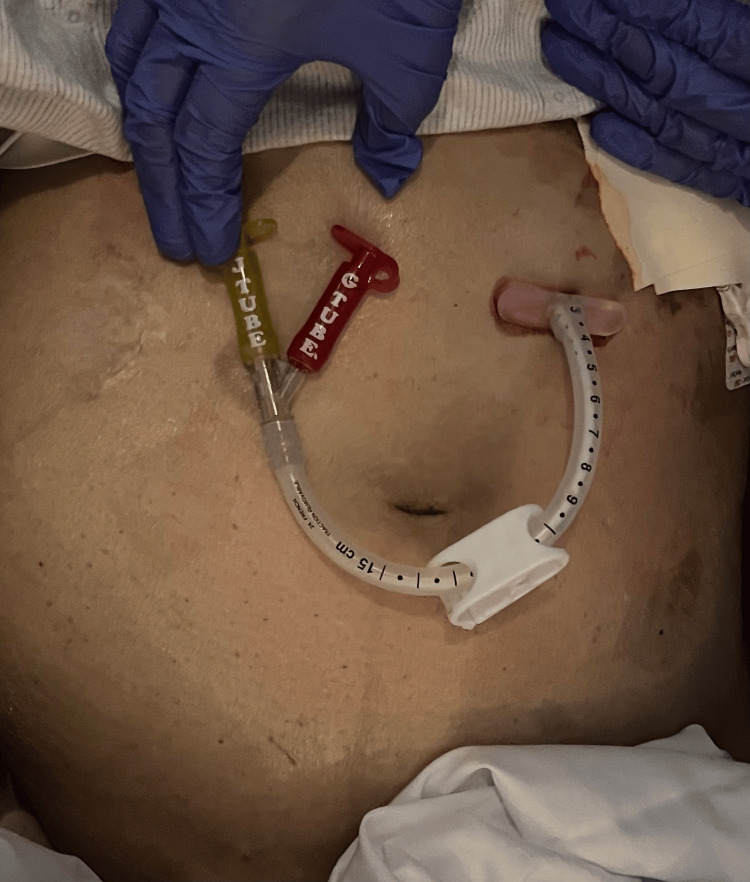
External view of the successfully placed gastrojejunostomy tube

**Figure 17:**
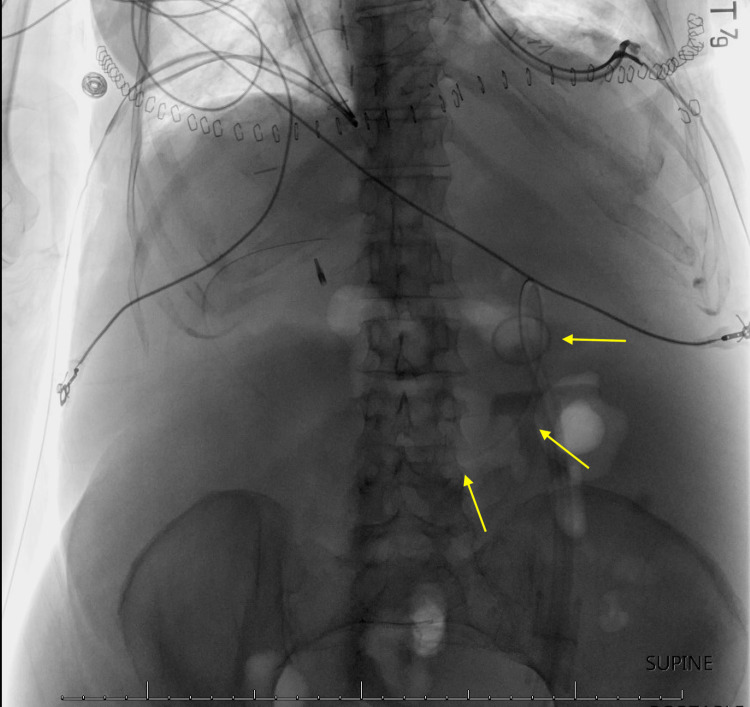
Abdominal X-ray confirming gastrojejunostomy tube placement (yellow arrows)

## Discussion

Indications for PEGJ placement include the inability to tolerate gastric feeding (typically due to severe gastroparesis, recurrent aspiration, or symptomatic gastroesophageal reflux), the need for long-term (>1 month) postpyloric enteral nutrition, the requirement for jejunal medication delivery, and the need for simultaneous gastric decompression and jejunal feeding [8,9]. Lung and heart transplant recipients are at particularly high risk of developing gastroparesis in the post-transplant period due to inadvertent vagal nerve injury or concurrent vagotomy performed during transplantation. Therefore, a gastrojejunostomy tube may be considered to optimize enteral feeding and reduce aspiration risk when long-term enteral nutrition is required. Gastrojejunostomy tube placement can be performed by experienced surgeons, gastroenterologists, or interventional radiologists. Several placement techniques exist, with the most common being the per-oral pull technique followed by placement of a jejunal extension through the PEG, as was performed in this case. The use of fluoroscopy is not necessary for endoscopic PEGJ placement. Absolute contraindications to PEG tube placement include partial or total gastrectomy, active intra-abdominal infection or sepsis, hemodynamic instability, and coagulopathy [10]. Ascites, morbid obesity, and peritoneal carcinomatosis are relative contraindications [10].

Percutaneous feeding tube placement is generally well tolerated; however, several major complications may occur, including bleeding, bowel perforation, or injury to adjacent structures [11]. There are no published data specifically addressing complication rates of percutaneous endoscopic gastrostomy or gastrojejunostomy in patients with SI, due to the rarity of the condition. In the general population, PEG and PEGJ are considered safe, with major complication rates typically ranging from 0.2% to 2% [12-15].

We encountered several challenges during the procedure due to the unusual anatomy. First, the optimal site for gastrostomy tube placement was along the lesser curvature of the stomach, as usual; however, it appeared on the right side of the endoscopic view and the left side of the patient’s abdomen. Accurate site identification can be confirmed with one-to-one finger indentation. The distance and path from the gastrostomy site to the pylorus were relatively straight. However, if the gastrostomy site is located farther from the pylorus, the tube may form a significant intragastric loop during the procedure, limiting advancement into the jejunum. Grasping the gastrostomy tube with rat-tooth forceps and advancing it toward the pylorus under endoscopic guidance can help reduce gastric looping and facilitate deeper jejunal positioning. Second, the reversed orientation and sharp angulation of the duodenum required cautious advancement of the endoscope to avoid excessive force on the small bowel and potential mucosal injury. Third, the angulation and tortuosity require the endoscopist to be familiar with multiple techniques for advancing the jejunal extension tube. These include advancing the tube over a guidewire either sequentially or in increments, as well as direct grasping of the tube or a suture loop using devices such as a snare, forceps, or an endoclip. For example, incremental advancement of the guidewire followed closely by the jejunal tube allows frequent reassessment of sharp angulations, reducing the likelihood of the tube becoming caught on a mucosal fold or sharp turn. Dragging the tube with a suture loop offers a similar advantage; however, once the tube is in the desired position, the suture loop may become entangled in the teeth of the grasping device (e.g., biopsy forceps). Therefore, an endoclip is often preferred for this technique. Lastly, significant angulation and tortuosity may require extensive torque steering and predispose to loop formation, resulting in unstable scope positioning and increased operator strain. Transfer of the loop toward the umbilicus is a technique commonly used in colonoscopy, which was helpful in this case. These techniques are not unique to patients with SI; however, mirror-image anatomy makes them more challenging. Longer procedure times may therefore be expected.

PEGJ tube placement in patients with SI has not been previously described. Successful placement is achievable with appropriate anatomical awareness and technical adaptation. In this case, we highlighted mirror-image orientation and emphasized where alternative techniques may be particularly useful.

## Conclusions

In this case, we employed various techniques to achieve PEGJ placement in a patient with SI. Careful preprocedural anatomical assessment is recommended to account for possible vascular or organ anomalies. Left versus right lateral decubitus positioning, in addition to supine positioning, should also be considered. Finally, when faced with difficulty advancing the jejunal extension tube, we modified our techniques to avoid mucosal injury. These adjustments may improve cannulation success and reduce complications, as mirror-image anatomy can make standard approaches more challenging. In this novel case report of gastrojejunostomy tube placement in SI, operator adaptation to the reversed anatomy was beneficial, demonstrating that procedural success may depend on the endoscopist’s experience and willingness to adjust technique and positioning.
